# Tracking the Traveled Distance of Capsule Endoscopes along a Gastrointestinal-Tract Model Using Differential Static Magnetic Localization

**DOI:** 10.3390/diagnostics12061333

**Published:** 2022-05-27

**Authors:** Samuel Zeising, Lu Chen, Angelika Thalmayer, Maximilian Lübke, Georg Fischer, Jens Kirchner

**Affiliations:** Institute for Electronics Engineering, Friedrich-Alexander-Universität Erlangen-Nürnberg (FAU), Cauerstraße 9, 91058 Erlangen, Germany; lu.chen@fau.de (L.C.); angelika.thalmayer@fau.de (A.T.); maximilian.luebke@fau.de (M.L.); georg.fischer@fau.de (G.F.); jens.kirchner@fau.de (J.K.)

**Keywords:** static magnetic localisation, trajectory tracking, traveled distance, wireless capsule endoscopy

## Abstract

The traveled distance and orientation of capsule endoscopes for each video frame are not available in commercial systems, but they would be highly relevant for physicians. Furthermore, scientific approaches lack precisely tracking the capsules along curved trajectories within the typical gastrointestinal tract. Recently, we showed that the differential static magnetic localisation method is suitable for the precise absolute localisation of permanent magnets assumed to be integrated into capsule endoscopes. Thus, in the present study, the differential method was employed to track permanent magnets in terms of traveled distance and orientation along a length trajectory of 487.5 mm, representing a model of the winding gastrointestinal tract. Permanent magnets with a diameter of 10 mm and different lengths were used to find a lower boundary for magnet size. Results reveal that the mean relative distance and orientation errors did not exceed 4.3 ± 3.3%, and 2 ± 0.6${}^{\circ }$, respectively, when the magnet length was at least 5 mm. Thus, a 5 mm long magnet would be a good compromise between achievable tracking accuracy and magnet volume, which are essential for integration into small commercial capsules. Overall, the proposed tracking accuracy was better than that of the state of the art within a region covering the typical gastrointestinal-tract size.

## 1. Introduction

Capsule endoscopy is an established medical modality for the diagnosis of the entire gastrointestinal tract (GIT) [1]. The capsules are equipped with cameras and a light source to record a video as they are passively moved through the GIT by peristalsis within 8 to 12 h [2]. Commercial capsules take a couple of frames per second. Consequently, more than 50,000 images of the GIT are recorded, resulting in a challenging and time-consuming video review process for doctors [3]. For this purpose, the automatic detection of abnormalities [4] within the GIT could increase the efficiency of diagnosis with capsule endoscopy. The efficiency and reliability of both the manual and automatic detection of abnormalities, and surgery planning would be even more improved if the traveled distance and orientation of the capsule were available for each video frame. In commercial capsule endoscopy systems, localisation accuracy of approx. 40 mm was reported [5], whereas orientation cannot yet be determined. Nevertheless, considering that commercial capsules measure up to 33 × 12 mm (length × diameter), the localisation accuracy of commercial systems is insufficient, since the error is larger than the capsule dimensions. In addition, the accurate orientation of the capsule should be tracked to determine the movement direction, which enables distinguishing between lateral and longitudinal movements of the capsule within the GIT and the rotations of the capsule. Against this background, many researchers are working towards the accurate localisation of capsule endoscopes, and the highest accuracy was reported when video-based and magnetic localisation was used [6,7].

In video-based approaches, differences in consecutive frames are employed to estimate the relative displacement and rotation of the capsule [3,8,9,10]. Recently, Iakovidis et al. (2019) [9], and Vedaei et al. (2021) [10] proposed the video-based tracking of the traveled distance of a capsule. The main advantage of video-based tracking is that it is independent of a static localisation reference. In both studies, the relative distance error for a capsule being moved at a constant speed along linear trajectories did not exceed approx. 6%. However, the peristalsis of the intestinal walls interferes with the capsule movement in the captured images, which was not considered in [9,10] since no in vivo experiments were conducted. Moreover, a linear trajectory does not reflect the complex and winding human GIT.

In magnetic-field-based approaches, a permanent magnet is assumed to be integrated into capsules with lengths of approx. up to 33 mm and diameters up to 12 mm. By measuring the magnetic field with a sensor array attached to the abdominal surface, the position and orientation of the capsule are estimated relative to the array. To the best of the authors’ knowledge, in state-of-the-art magnetic localisation approaches, the 3D coordinates of the capsule are usually determined, but not its traveled distance [11,12,13,14,15]. The main advantage of magnetic-field-based localisation is that the interaction of static and quasistatic magnetic fields within the human body is negligible, since its relative permeability is approx. 1 [16].

In our previous work [17,18], a differential static magnetic localisation method for capsule endoscopes was proposed. Simulations [17] and experiments [18] showed that the absolute coordinates and orientation of a permanent magnet of size 10 × 10 mm can be determined with accuracy of approx. 3 mm and 2${}^{\circ }$, respectively. In addition, the magnet was continuously moved along a short linear 3D trajectory, and the traveled distance of the magnet was simultaneously tracked with a relative distance error of 3.1%, and its orientation with an error of 2.7${}^{\circ }$. However, the experiment was conducted in a relatively small region of approx. 100 × 100 × 30 mm that did not reflect the typical size of a GIT. Furthermore, the GIT is much more complex than a linear trajectory. In addition, considering the dimensions of typical commercial capsules (3 × 12 mm), the volume of the permanent magnet should be as small as possible. To the best of the authors’ knowledge, Wang et al. (2019) [19] were the first to propose tracking the traveled distance of capsule endoscopes using static magnetic localisation. A permanent magnet whose size was not stated was tracked along a 3D curved trajectory with close distance to a planar array. Nevertheless, it could not be assumed that the distance between capsule and array would be relatively short during the entire diagnostic procedure. Moreover, Su et al. (2017) [20] revealed that using planar sensor arrays for static magnetic localisation is impracticable since localisation accuracy highly depends on the distance between array and magnet.

Therefore, in this study, the differential static magnetic localisation method proposed by the authors in [17,18] was applied to track the traveled distance and the orientation of permanent magnets that were moved along a 3D curved trajectory within a region that approx. covered the typical size of a GIT. The aims of this study were to keep the relative distance error of the traveled distance below 5%, since the typical length of the GIT is approx. 8 m [21], and the orientation error below 5${}^{\circ }$ to be able to track capsule rotations and its movement direction. Furthermore, a lower boundary for the permanent magnet volume was determined to accomplish these aims, which is crucial for possible integration into commercial capsules where space is very limited.

## 2. Methods

In the following, the concept of differential static magnetic localisation is explained. Subsequently, the used localisation setup for evaluating of the tracking method is described. Lastly, the evaluation procedure of this study is outlined.

### 2.1. Differential Static Magnetic Tracking

In static magnetic localisation, a permanent magnet is assumed to be integrated into capsules with a typical size of 33 × 12 mm. Generated magnetic flux density $B_{\mathrm{mag}}$ at an observed point $P_{o}$ with Euclidean distance *R* to the magnetic centre can be analytically described. This analytical formula is the standard magnetic dipole model and applies when distance *R* is much higher than the largest dimension of the magnetic source. The model is defined as follows [22]:

(1) $$B_{\mathrm{mag}}\left(P_{o}\right)=\frac{\mu_{0}\mu_{r}M_{0}l\pi k^{2}}{4\pi }\left(\frac{3\langle O_{0},R\rangle R}{{∥R∥}_{2}^{5}}-\frac{O_{0}}{{∥R∥}_{2}^{3}}\right),$$

where $O_{0}$ corresponds to the orientation of the magnetic source, $\mu_{0}$ is the magnetic permeability in vacuum ($4\pi \times 10^{-7}$ H/m), and $\mu_{r}$ refers to the relative permeability of human tissue, which is approx. 1 [16].

For static magnetic localisation, a sensor array is assumed to be arranged outside the body to measure the magnetic flux density at known reference positions. However, the authors in [23], revealed that, at the sensor array, the geomagnetic flux density is of the same order as that of the generated magnetic flux density. Thus, in [17,18], a differential static magnetic localisation method was proposed by the authors to compensate for this interference. The differential method groups the sensor array into pairs of equally oriented sensors. Consequently, the homogeneous geomagnetic flux density cancels out when the difference between the measured and the analytical values ΔB for the sensors of pairs is subtracted.

(2) $$\Delta B_{\mathrm{pair}}=\Delta B_{\mathrm{sensor}1}-\Delta B_{\mathrm{sensor}2}.$$

Deriving $\Delta B_{\mathrm{pair}}$ for all pairs, resulting in a K·3×6 nonlinear equation system, where *K* is the number of pairs. The nonlinear equation system is solved for the position and orientation relative to the sensor array applying the well-established Levenberg–Marquardt (LM) algorithm to minimise error function $\epsilon_{\mathrm{diff}}$

(3) $$\epsilon_{\mathrm{diff}}=\sum_{i=1}^{K}{∥\Delta B_{{\mathrm{pair}}_{i}}∥}_{2}.$$

**Figure 1:**
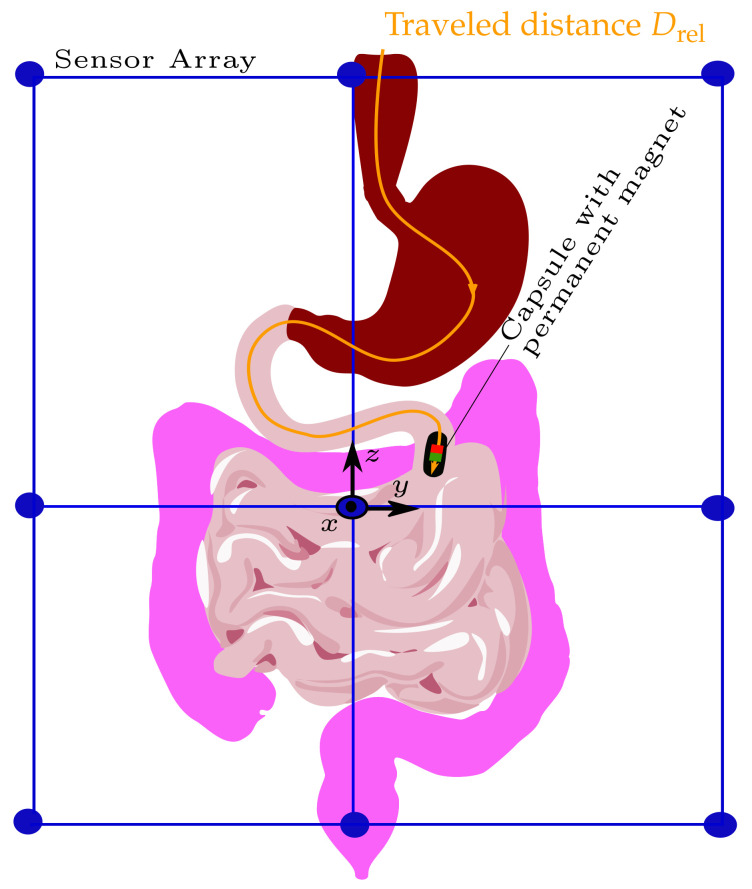
Schematic of the system design using the differential method to track the traveled distance of a capsule endoscope within the gastrointestinal tract.

With positions $P_{i}$ of the magnet for the *i*-th sample, total traveled distance $D_{\mathrm{rel}}$ is determined by

(4) $$D_{\mathrm{rel}}=\sum_{i=1}^{M}{∥P_{i+1}-P_{i}∥}_{2},$$

where *M* is the total number of samples, i.e., successive magnet positions. The schematic of tracking the traveled distance of a capsule endoscope as it passes the GIT is depicted in Figure 1.

### 2.2. Experimental Setup

**Figure 2:**
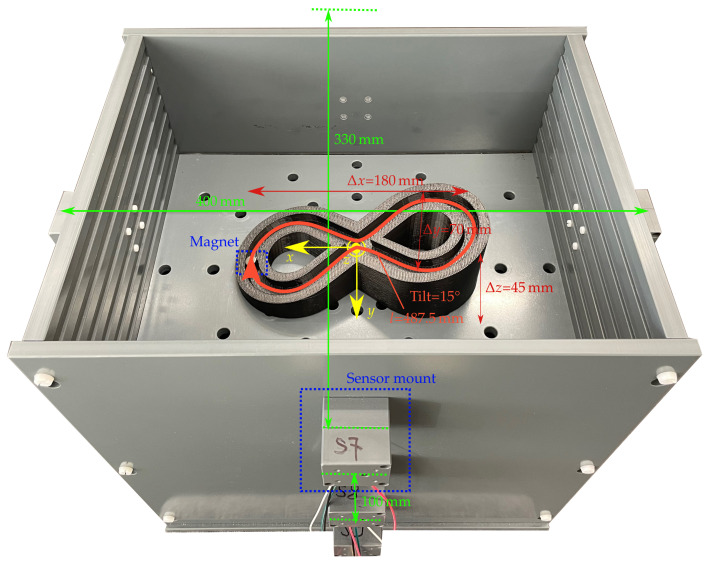
Localisation setup with the 12 sensors centered within the sensor mounts. The coordinate system of the setup is in its centre. The printed trajectory with the 10 × 10 mm magnet was exemplarily placed at the z=0 mm plane.

Figure 2 shows the setup for validating differential magnetic tracking along a 3D curved trajectory within a region that approx. covers the typical size of a GIT.

Moreover, the setup enables the use of different magnets for possible integration into commercial capsules. The setup comprises 12 LSM303D magnetic sensors, which are arranged on three elliptical rings with diameters of (400 × 330 mm) and are placed at a distance of 100 mm to each other in the *z* direction. Consequently, the setup covers the size of a typical abdomen. The sensor array is intended to be integrated with a wearable system such as a vest in the daily life of a patient.

The interintegrated circuit (I2C) bus was employed to connect the LSM303D sensors with an Arduino board. The full-scale range and sampling frequency of the sensors were set to ±800 µT and 50 Hz, respectively, resulting in a resolution of 32 nT/LSB. The measured signal was averaged over three samples. The magnetic sensors were calibrated for soft and hard magnetic distortions using the ellipsoid fitting algorithm proposed in [24]. As static magnetic sources, three neodymium N52 cylindrical permanent magnets with a diameter of 10 mm and lengths of 3, 5, and 10 mm with an axial magnetisation of approx. 1150 kA/m were used.

Moreover, a 3D-printed trajectory with a total travel length of approx. l= 487.5 mm and a tilt angle of approx. 15${}^{\circ }$ was used, and the *z* position of the trajectory was adjustable with a step size of 20 mm.

### 2.3. Evaluation Procedure

**Figure 3:**
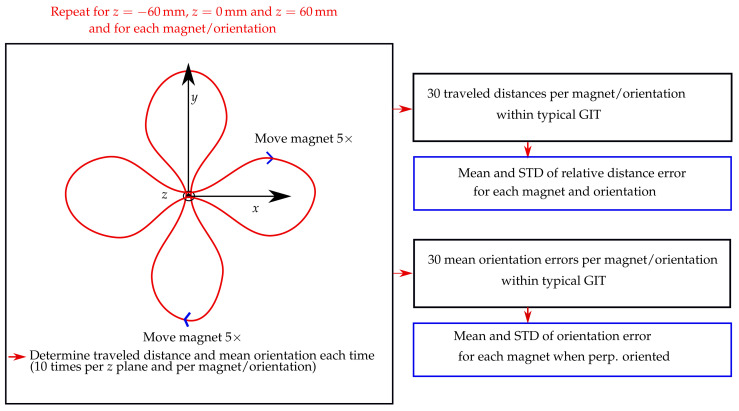
Schematic of the evaluation procedure for calculating the mean and standard deviation (STD) of the relative distance error and orientation error for each magnet, and its corresponding orientation.

The schematic of the evaluation procedure of this experimental study is shown in Figure 3. The trajectory was placed in three different *z* planes of the localisation setup, namely, 60, 0, and 60 mm. Moreover, the vertical displacement of the magnet as it traveled along the trajectory was approx. 49 mm. For each *z* plane, the trajectory was in the *x* and *y* orientations. Consequently, the stability of the traveled distance of the magnet was evaluated in a region of approx. 180 × 180 × 150 mm, which approximately covered the size of a typical GIT.

The magnets were moved with direct contact to the outer wall along the entire trajectory within 40 s, while their magnetisation was perpendicular with the moving direction. Furthermore, for the 10 mm long magnet, magnetisation was also parallel with the moving direction. In contrast, for the 3 and 5 mm long magnets, it was impossible to precisely move them along the outer wall when in parallel with the moving direction. The magnets were moved five times along the trajectory for each trajectory orientation and *z* plane, resulting in 10 measurements per *z* plane and 30 measurements in total for each magnet and magnet orientation.

Subsequently, relative distance errors regarding the actual trajectory length of 487.5 mm were derived for the 30 measurements per magnet and magnetisation orientation. Next, the mean and standard deviations (STD) concerning these 30 relative distance errors were derived for each magnet and magnetisation orientation. Moreover, when the magnetisation of the magnets was perpendicular with the moving direction, the mean orientation error along the trajectory was derived for each of the 30 measurements per magnet using the actual tilt angle of the trajectory (15${}^{\circ }$) as a reference. However, for the measurements where the magnetisation was in parallel with the moving direction, the orientation error of the magnet along the trajectory was not determined, since no reference for the magnet orientation along its path was available. Lastly, the mean and STD values of the orientation error concerning the 30 mean orientation errors per magnet were derived.

## 3. Results

**Figure 4:**
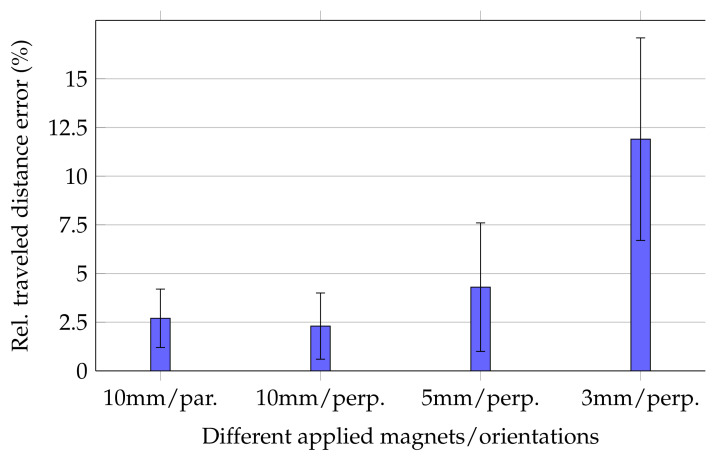
Mean and standard deviation values for relative traveled distance errors for different magnets and orientations. For the 5 and 3 mm long magnets, cases where the magnetisation was in parallel with the moving direction were not conducted.

Relative errors of the traveled distance for moving the magnets along the trajectory are shown in Figure 4.

**Figure 5:**
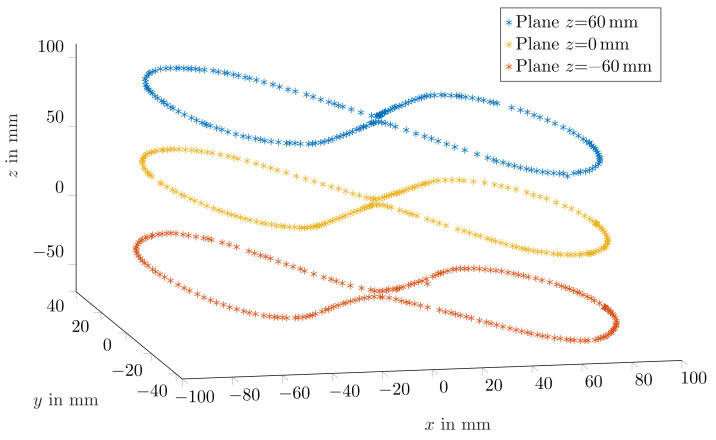
Representative measured trajectories using the 10 × 10 mm magnet while its magnetisation was perpendicular to the movement direction.

For the 10 mm long magnet, the mean relative traveled distance errors for cases in which magnetisation was perpendicular and in parallel with the moving direction were 2.3% ± 1.7% and 2.7% ± 1.5%, respectively. Consequently, since the margins of error for both magnetisation orientations of the 10 mm long magnet were overlapping, tracking accuracy was expected to be stable for different magnet orientations. Moreover, Figure 5 depicts three representative trajectories for the three *z* planes when magnetisation was perpendicular with the moving direction. The measured trajectories were in good agreement with the physical trajectory. However, since the magnet was moved by hand, the distance between successive points was not uniform.

For the 5 and 3 mm long magnets, relative errors were 4.3% ± 3.3% and 11.9% ± 5.2%, respectively. Comparing these relative errors with each other and those of the 10 mm long magnet indicated that the relative error considerably increased for smaller magnets.

The high standard deviation for the 3 mm long magnet was the reason for the relative high relative distance error using the smallest magnet. In contrast, the standard deviations for the 5 and 10 mm long magnets are acceptable since their mean relative distance error was sufficiently low.

**Figure 6:**
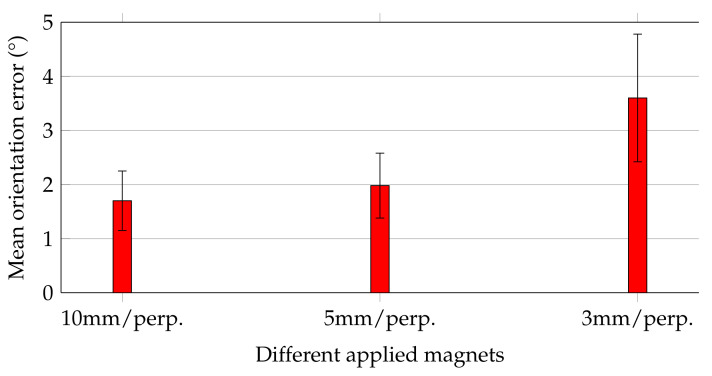
Mean and standard deviation values of orientation errors for magnets along the trajectory in perpendicular orientation to the moving direction.

Figure 6 shows the mean orientation errors of the magnets while they were moved along the trajectory, and their magnetisation was perpendicular with the moving direction.

Orientation errors followed the same trend as that of the relative distance errors for the three magnets. For the 10 and 5 mm magnets, the mean orientation errors agreed well and did not exceed 2${}^{\circ }$ ± 0.6${}^{\circ }$. In contrast, for the 3 mm magnet, the mean orientation error was increased by approx. a factor of 2 to 3.6${}^{\circ }$ ± 1.2${}^{\circ }$. Considering that this study aimed to track the orientation of capsule endoscopes with accuracy of at least 5${}^{\circ }$, all applied magnets would be sufficient for that purpose.

## 4. Discussion

In this study, a tracking system for capsule endoscopes was proposed, and the aims of this work were that the traveled distance of the capsule be tracked with a mean relative distance error below 5% and a mean orientation error below 5${}^{\circ }$ within a GIT of typical size. Results reveal that these aims were successfully accomplished when permanent magnets of 5 and 10 mm lengths were integrated into the capsules. Consequently, the 5 mm long magnet is a good compromise between accuracy and magnet volume, which are crucial for integration into commercial capsules with approx. sizes of 33 × 12 mm. In contrast, the 3 mm long magnet resulted in considerably higher tracking errors; thus, a magnet of size 5 × 10 mm was regarded to be the lower boundary. Moreover, results reveal that tracking accuracy was stable for different magnet orientations, which is crucial since the capsule is arbitrarily orientated relative to the sensor array in real applications. Overall, the proposed study demonstrated that the differential static magnetic method is suitable for capsule endoscopy. Nevertheless, the experimental setup was rigid, and the method has not yet been tested in in vivo experiments on a patient. Integration with a wearable system such as a vest is challenging since the sensors’ positions and orientations may vary in the daily life of a patient, which results in higher tracking errors than those in the relatively simple experimental study.

In the following, the proposed differential method is compared with state-of-the-art tracking methods for capsule endoscopes. Table 1 gives an overview of existing methods in comparison with the presented work.

In state-of-the-art static magnetic localisation methods with geomagnetic compensation, to the best of the authors’ knowledge, the absolute coordinates and orientation concerning the sensor array are determined instead of the traveled distance [11,12,13,14]. Therefore, these approaches could not be directly compared with the proposed relative localisation method. In [19], a permanent magnet of unstated size was moved along a 3D trajectory with a length of 840 mm. The standard static magnetic localisation method without a geomagnetic compensation method was applied to track the traveled magnet distance. However, this method is only reliable when the patient remains in a fixed position and orientation during the entire diagnostic procedure. Since this procedure lasts for several hours, using this method is impracticable. In their study, a relative error of 5.7% was reported. However, the trajectory was close to that of the used planar sensor array, which was beneficial for the results. A planar sensor array has limited sensing range, and thus is not suitable for the application on a patient to track capsule endoscopes within the entire GIT. The capsule orientation was also not determined, which would be required for diagnosis. In [18], we tracked a magnet of size 10 × 10 mm that was moved along a 109 mm long linear 3D trajectory. A 3D sensor array covering the typical size of an abdomen was used for tracking the magnet. The mean relative distance error of the magnet along the linear trajectory was approx. 3.4%. Thus, the mean relative distance errors of the 10 mm long magnet in the present study using the 487.5 mm long nonlinear trajectory was slightly reduced to approx. 2.7%. Therefore, an accumulation of the mean relative distance error for a longer trajectory when a 10 mm long magnet was used was not observed, which is highly relevant since the typical GIT is approx. 8 m long [21].

Moreover, in [9], the traveled distance of a capsule while it was moved along a 200 mm long linear trajectory was determined with a relative distance error of approx. 6% using video-based tracking. In [10], four sidewall cameras and an inertial unit that had a total volume that exceeded the typical size of a commercial capsule were used to track the traveled distance. The capsule was moved along a 600 mm long linear trajectory with approx. 3.7% accuracy. However, the reported results of the video-based approaches were achieved using linear trajectories, which insufficiently reflect the complex GIT. Moreover, no in vivo experiments have been conducted in video-based tracking, which is crucial to evaluate the impact of the peristalsis of intestine walls on accuracy.

Compared to existing state-of-the-art methods for tracking the traveled distance of capsule endoscopes, in the proposed study, to the best of the authors’ knowledge, for the first time, both traveled distance and orientation were estimated. Moreover, in video-based methods, the capsules were moved at constant speed along linear trajectories that do not sufficiently reflect the complex human GIT. The only video-based approach where a moderately lower relative distance error than that of the presented study was reported was in [10]. However, here, four sidewall cameras and an IMU were used that exceeded the typical size of a commercial capsule endoscope. In contrast, the proposed tracking method occupied only approx. 10.5% of the volume of commercial capsules. Moreover, compared to the static magnetic tracking approach proposed in [19], a 3D sensor array with higher spatial diversity resulted in high tracking accuracy within the typical size of a GIT that was used in the presented study. The proposed method is also applicable in the dynamic daily life situations of a patient since it offers geomagnetic compensation. Overall, the differential static magnetic localisation method is superior to state-of-the-art methods, and the next step towards tracking the traveled distance and orientation of capsule endoscopes in the daily life of a patient.

The main limitations of the study were that no prototype capsule with an integrated magnet has been considered that was swallowed by real subjects. In addition, the proposed localisation system is not yet ready for integration with a wearable system, since a rigid setup was considered, and a more flexible array is desired. Furthermore, the trajectory was much shorter than that of the typical human GIT; for the 5 mm magnet, if the relative distance error is stable for longer trajectories must be investigated. Another limitation of this study was that the magnet orientation was not yet used for enhancing the accuracy of the traveled distance. With the precise moving direction of the capsule, e.g., lateral movement of the capsule within the small intestine could be detected and not be considered for the total traveled distance. Moreover, magnetic distortions from nearby ferromagnetic objects were not investigated, which could have resulted in higher relative distance and orientation errors.

## 5. Conclusions

This study demonstrated that differential static magnetic localisation is well-suited for capsule endoscopes when a permanent magnet of at least 5 mm length and 10 mm diameter is integrated. The setup to evaluate the method was rigid, and in a real application on the patient, the method must be implemented in a wearable system. In future work, a prototype capsule with an integrated magnet and a wearable system should be designed and tested on subjects to investigate the performance of the proposed tracking system under more realistic conditions.

## Figures and Tables

**Table 1:** Comparison with state-of-the-art capsule endoscopy tracking methods. The occupied space is specified with respect to the assumed typical size of commercial capsules of 33 × 12 mm (length × diameter).

	Method	Trajectory	Occ. Space (%)	Rel. Error (%)	Or. Error (${}^{\circ }$)
This work:					
10 mm magnet	Diff. static magnetic	Curved 3D (487.5 mm)	21.0	2.7	1.7
5 mm magnet	Diff. static magnetic	Curved 3D (487.5 mm)	10.5	4.3	2.0
3 mm magnet	Diff. static magnetic	Curved 3D (487.5 mm)	6.3	11.9	3.6
State of the art:					
[19] (2019)	Static magnetic	Curved 3D (840 mm)	not stated	5.7	-
[18] (2021)	Diff. static magnetic	Linear (109 mm)	21.0	3.1	2.7
[9] (2019)	Video	Linear (200 mm)	0	6.0	-
[10] (2021)	Video/Inertial unit	Linear (600 mm)	>100	3.7	-

## Data Availability

Matlab code and data are available from the corresponding author upon request.
